# Special Departments at Small Hospitals

**Published:** 1896-04-04

**Authors:** 


### The Institutional Workshop.

### SPECIAL DEPARTMENTS AT SMALL HOSPITALS.

The resignation of four members of the medical staff of the Leith Hospital draws attention to the affairs of that institution, but at the same time it raises issues which are of much wider application. It appears that in November last an operation was performed in the hospital by a gentleman who was not a member of the staff. It was the opinion of the board that this should not have been done, at any rate without taking the opinion of the consulting surgeon to the institution, and the board being communicated with. The question also was thus raised whether the staff of the hospital was sufficient for the work, and at the December meeting it was resolved that the staff should be supplemented by the appointment of a gynæcologist, an oculist, and a pathologist. Another question also came up as to whether the appointments should be confined to men practising in Leith, or should be open to those resident in Edinburgh, and it was decided to leave this open.

At the January meeting the resignations of four of the staff were received, no reason being given, and the vacancies thus caused were filled up. How far this course was in accordance with the rules of the institution we do not know, but it seems fairly clear that those medical men who sent in resignations and gave no reasons can have no *locus standi* in regard to any subsequent proceedings, and in regard to appointing outside men it must be remembered that Leith is but a part of Edinburgh, with no dividing line, and that Edinburgh is one of the greatest medical schools in the world, and that it would seem absurd to deny to the people of Leith the advantage of the skill and experience which accumulates round so important a medical centre.

While saying this, however, we do not wish it to be understood that we look with favour upon the plan of small hospitals in outlying districts deputing their special work to specialists from great towns, who may come down at stated intervals, see special cases, do special operations, and take their special knowledge back with them to the great towns where they live. We think that every hospital, small as much as large, has a duty to those outside its walls as well as to those within, and that no hospital completely fulfils its mission unless it becomes a centre of quite up-to-date medical knowledge and surgical skill, and thus becomes a benefit to the district in which it stands, as well as to the sick who enter it. Now this can never be the case if the local men are asked to do the humdrum work, and specialists from distant places are called in to take charge of the interesting cases. Still some arrangement should be made by small hospitals, in places where resident specialists would be out of the question, for patients obtaining the benefit of the best and most recent knowledge on the subject if their cases are out of the common run. This, however, should be done, not by bringing the specialist to the patient, but by arrangement between small hospitals in country districts, and great or special ones in large and populous centres for the treatment of such cases as it may be considered desirable to transfer from one to the other.

Even in the most out of the way districts there will always be cases such as cataracts, and in fact most kinds of operative ophthalmic work which should be sent to specialists, but such cases are never urgent as to time, and they could always be transferred to other hospitals if proper arrangements were made for the purpose. The same holds good in regard to certain abdominal operations. But it would be unjust to any district that, because a few chronic cases can be dealt with by large or special institutions, the surgeons of that district should be deprived of experience in that class of diseases, as they would be were such cases handed over to the care of peripatetic specialists. No part of the country can be considered properly provided with the appliances of civilisation unless it has among its resident medical men individuals able to deal properly with the most urgent cases and to do with complete and metropolitan perfection all operations of emergency. Chronic things may be left to great centres of clinical teaching, but a local hospital fails in its mission unless it draws around it a group of men who are thoroughly capable of dealing in the very best possible manner with the most urgent surgical work, such as traumatic aneurisms, perforating gastric ulcers, intestinal obstructions, extra uterine fœtations, and all forms of accident. Every acre in England, Scotland, and Ireland is within an easy journey of the great medical centres, and if proper inter-hospital arrangements were made, there need be nothing derogatory to the dignity of the medical officer of the small hospital in transferring his chronic patients to the large one in case of difficulty. This would be far better than the plan which we know exists in some of the smaller hospitals of having outside specialists attached to them in certain departments, for the intrusion of such men greatly tends to lessen the responsibility of the resident staff, and takes away from the utility of the small hospital as a centre of medical knowledge of a type higher than would exist but for the opportunities it should offer to the medical men of the district.

